# Occurrence, characterization, and potential predictors of verotoxigenic *Escherichia coli*, *Listeria monocytogenes*, and *Salmonella* in surface water used for produce irrigation in the Lower Mainland of British Columbia, Canada

**DOI:** 10.1371/journal.pone.0185437

**Published:** 2017-09-27

**Authors:** Justin Falardeau, Roger P. Johnson, Franco Pagotto, Siyun Wang

**Affiliations:** 1 Department of Food, Nutrition, and Health, The University of British Columbia, Vancouver, British Columbia, Canada; 2 Public Health Agency of Canada, Guelph, Ontario, Canada; 3 Listeriosis Reference Service, Bureau of Microbial Hazards, Health Canada, Ottawa, Ontario, Canada; University of Campinas, BRAZIL

## Abstract

Produce has become a major source of foodborne illness, and may become contaminated through surface water irrigation. The objectives of this study were to (i) determine the frequency of verotoxigenic *E*. *coli* (VTEC), *Listeria monocytogenes*, and *Salmonella* in surface waters used for irrigation in the Lower Mainland of British Columbia, (ii) assess the suitability of fecal coliforms and generic *E*. *coli* as hygiene indicators, and (iii) investigate the correlations of environmental factors with pathogen occurrence. Water samples were collected semi-monthly for 18 months from seven irrigation ditches across the Serpentine and Sumas watersheds. VTEC colonies on water filters were detected using a verotoxin colony immunoblot, and the presence of virulence genes *vt1* and *vt2* was ascertained via multiplex PCR. Detection of *L*. *monocytogenes* and *Salmonella* was completed using standard, Health Canada Compendium of Analytical Methods. Fecal coliforms and generic *E*. *coli* were enumerated by 3M™ Petrifilm™ and filtration methods, and meteorological and geographic data were collected from government records. VTEC, *L*. *monocytogenes*, and *Salmonella* were detected in 4.93%, 10.3%, and 2.69% of 223 samples, respectively. *L*. *monocytogenes* occurrence was greatest in the Serpentine watershed (χ^2^; p < 0.05), and was most common during the winter and fall (Fisher exact test; p < 0.05). Site dependence of VTEC and *Salmonella* occurrence was observed within watersheds (Fisher’s exact test; p < 0.10). Pathogen occurrence correlated with fecal coliform counts (r = 0.448), while VTEC occurrence also correlated with precipitation over the five days before sampling (r = 0.239). The density of upstream livestock correlated with VTEC (r_s_ = 0.812), and *L*. *monocytogenes* (r_s_ = 0.841) detection. These data show that foodborne pathogens are present in the waters used for irrigation in the Lower Mainland of British Columbia, but their frequency may depend on spatial and temporal factors.

## Introduction

Foodborne illness continues to be an issue for consumers. In addition to health costs, foodborne illnesses can also lead to an economic burden due to lost productivity and wages among those affected [1]. Though foodborne pathogens, such as *Listeria monocytogenes*, *Salmonella*, and verotoxigenic *Escherichia coli* (VTEC), are generally associated with meat and dairy products, produce has been observed to be a major source of foodborne illness: produce accounted for more foodborne illnesses and outbreaks than any other food category in the United States between 2004 and 2013 [2], and led to 27 outbreaks in Canada between 2001 and 2009 [3]. Notable outbreaks include the 2011 outbreak of VTEC O104:H4 from sprouts, resulting in 4,068 cases with 908 cases of hemolytic uremic syndrome and 50 deaths in Europe, along with six cases in the United States and one case in Canada [4,5]; *Salmonella* Newport and *Salmonella* Poona on cucumbers causing 275 and 907 cases, including 7 deaths, respectively in the United States [6,7]; and most recently, an outbreak of *L*. *monocytogenes* involving prepackaged salads resulting in 19 illnesses and one death in the US [8], and 14 illnesses and three deaths in Canada [9].

Contaminated irrigation water is a likely source for fresh produce contamination. The use of poor quality water for irrigation has been correlated with increased incidence of foodborne infections [10], and experiments have specifically shown the effective transmission and internalization of *E*. *coli* in lettuce through spraying with contaminated water [11]. Once attached, the bacteria are able to survive for long periods, and may not to be removed through washing [12], thus leading to considerable risks associated with any produce consumed raw, such as leafy greens and sprouts.

Water testing for the presence of foodborne pathogens is too expensive and time consuming for routine analysis, therefore water quality assessment and regulation has been based on the concentrations of microbial indicators. These are bacteria that are non-pathogenic, but are accepted to correlate with the presence of foodborne pathogens [13]. Water quality guidelines in Canada recommend fewer than 1,000 total coliforms and 100 fecal coliforms (FC) per 100 ml of water for surface waters used in crop irrigation [10,14]. Some provinces have also instituted their own recommendations. In British Columbia, the Ministry of the Environment recommends fewer than 200 FC per 100 ml, and fewer than 77 generic *E*. *coli* per 100 ml in all waters used for irrigation of produce [15]. Currently in Canada, however, no water quality standards for irrigation water have been legislated. In the United States, legislation soon to be enacted under the Food Safety Modernization Act (FSMA) will require the monitoring of trends of generic *E*. *coli* to estimate water quality [16]. The law will require a geometric mean below 126 CFU of *E*. *coli* per 100 ml across 20 consecutive samples, with a statistical threshold value no greater than 410 CFU of *E*. *coli* per 100 ml. Furthermore, no *E*. *coli* can be present in any water used during or after harvest, or to irrigate sprouts. The use of fecal indicators as predictors for the presence of pathogenic bacteria has shown limited success according to recent studies. An investigation by Pachepsky et al. [17] reviewed 81 datasets comparing concentrations of FC and *E*. *coli* in surface waters with the concentrations of one or more foodborne pathogenic organisms and only found a significant relationship in 35% of the cases reviewed. Moreover, according to a study in California, the concentration of *E*. *coli* was found to be not associated with the occurrence of VTEC O157 or *Salmonella* in either sediment or in surface water [18].

Alternatively, the occurrence of foodborne pathogens in water samples has been observed to correlate with various environmental factors such as landscape, weather, and season. A cross-Canada survey of surface waters found *Salmonella* to occur more frequently in areas affected by animal agriculture [19]. Similarly, recovery of *L*. *monocytogenes* from surface water was correlated with proximity to the nearest upstream dairy farm in Ontario [20], and proximity to land used for pasture in New York State [21]. With respect to weather, higher precipitation was shown to correlate with higher occurrence of VTEC O157 [22] and *Salmonella* [23] in Georgia. Similarly, precipitation three days prior to sampling was significantly correlated with the occurrence of VTEC O157 in Alberta [24], VTEC in British Columbia [25], and *Salmonella* in New York State [26]. Finally, pathogen occurrence has been observed to change over season, but not always consistently in different areas. The occurrence of VTEC O157 was observed to be higher during the summer months in Alberta [27] and Georgia [22], but was more common during the winter in California [28]. In British Columbia, VTEC of any serotype was also most common during the winter [25]. More consistency has been observed for *L*. *monocytogenes*, which is most prevalent during the winter [29,30], and *Salmonella*, which has been observed to be most common during the summer [23,24].

To date, only one study has investigated the occurrence of VTEC in the surface waters of the Lower Mainland of British Columbia [25], and none has investigated the occurrence of *L*. *monocytogenes* or *Salmonella*. Furthermore, no studies in the area have investigated the relative occurrences these three pathogens together, or studied any geographical and environmental sources affecting their occurrence. Therefore, the primary purpose of this study was to investigate the occurrence of VTEC, *Salmonella*, and *L*. *monocytogenes* in surface waters used for irrigation in the Lower Mainland of British Columbia, and to assess the usefulness of various predictors of their presence.

## Materials and methods

### Sample collection

**Fig 1:**
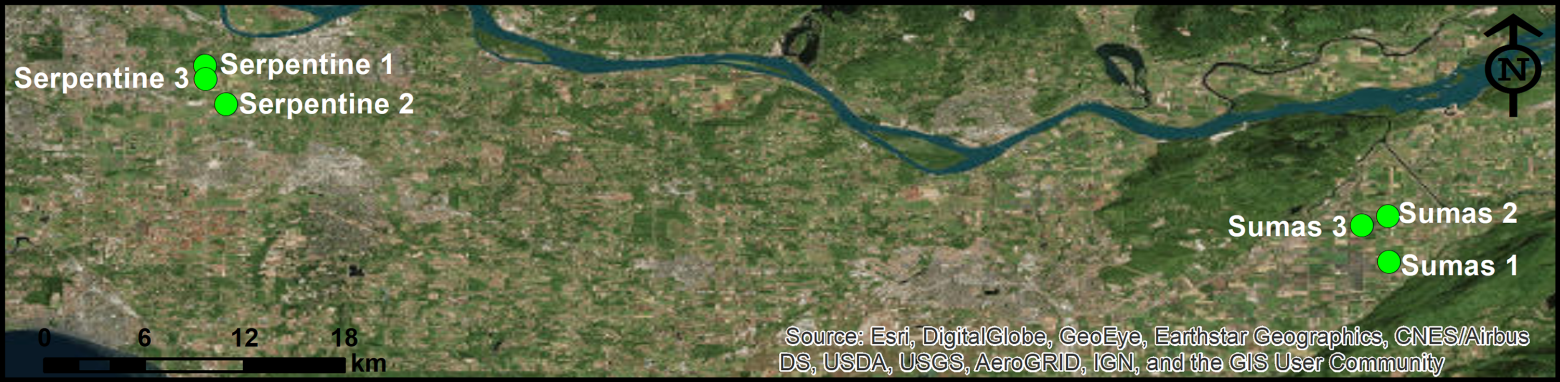
Sampling sites in the Serpentine and Sumas watersheds of the Lower Mainland of British Columbia, Canada.

Sampling sites were located in two distinct watersheds: The Serpentine watershed in the Cloverdale region of Surrey, BC, and the Sumas watershed on the Sumas prairie near Abbotsford, BC. Sample site locations are shown in Fig 1. Three surface water sites (*i*.*e*., ditch, creek, or stream) within each watershed were chosen which represented water adjacent to vegetable growing fields, with a fourth site (*i*.*e*., Sumas 1b) added to the Sumas watershed during the first summer of sampling. Sumas 1b was located approximately 30 meters down a side ditch attached to Sumas 1a and the two shared an upstream water source. For the sample sites in the Serpentine watershed, water samples were collected from the side of the road, and no special permission was required to collect the samples. The field study did not involve endangered or protected species. For the Sumas sites, the owner of the properties where the sample sites were located gave us specific permission to come onto the land to collect the samples. All sampling sites were directly connected (upstream or downstream) to source water used for irrigation of the adjacent produce growing operations. Irrigation was conducted during the growing season (approximately May to September) as necessary depending on rain fall.

Water samples were collected from each site once per month from February 2015 to April 2015, then twice per month until August 2016, with the exception of October 2015 when only one sample was collected from each site. Sampling began in February 2015 for Sumas 1a, March 2015 for Sumas 2 and Sumas 3, April 2015 for Serpentine 2, May 2015 for Serpentine 1 and Serpentine 3, and July 2015 for Sumas 1b.

Surface water samples were collected from the ditches in 532 ml Stand-Up Whirl-Pak® bags using a telescopic sampling pole designed to hold the Whirl-Pak® bags (Nasco, Fort Atkinson, WI). The sampling pole was rinsed thoroughly with 70% ethanol before and after sampling at each site. Five samples were collected at each site: two to be tested for *L*. *monocytogenes* and *Salmonella*, and two to be tested for VTEC. The fifth sample was used to measure the temperature of the sample water using an alcohol thermometer.

### VTEC detection, isolation, and characterization

The presence of VTEC in water samples was determined using a verotoxin immunoblot (VT-IB) method as previously described [25,31], with minor modifications. Briefly, 25 ml of each sample was vacuum filtered through a 0.45 μm Hydrophobic Grid Membrane Filter (HGMF; Neogen Corp., Lansing, MI). Each HGMF was then overlaid on a VT capture membrane and incubated for 18–24 hours at 37°C on modified tryptic soy agar (Becton Dickinson, Mississauga, ON) containing 1.5 g/L bile salts No.3, 10 μg/ml vancomycin and 10 μg/ml cefsulodin (Sigma, Oakville, ON) (mTSA-VC). The VT capture membranes were 82 mm diameter round 0.2 μm nitrocellulose filter membranes (Biotrace, Pall Life Sciences, Mississauga, ON) pre-coated with rabbit anti-VT antibodies reactive with all known variants of verotoxin (Public Health Agency of Canada, National Microbiology, Guelph, ON (NML Guelph)). The VT capture membranes were probed for the presence of VT using a mix of four mouse monoclonal anti-VT antibodies (2 μg/ml each; NML Guelph), followed by alkaline phosphatase-labelled anti-mouse IgG (0.02 μg/ml; Jackson ImmunoResearch Inc., West Grove, PA, USA) and visualized using the substrate nitroblue tetrazolium/5-bromo-4-chloro-3-indolyl phosphate (Mandel Scientific, Guelph, ON). Dark purple spots on the membrane indicating the presence of VT were used to identify the location of suspected VTEC isolates on the corresponding HGMF.

Up to eight suspected VTEC colonies were selected from the HGMF and streaked onto MacConkey agar (Becton Dickinson) and incubated at 37°C for 18–24 hours in preparation for confirmation of VT production using a VT enzyme linked immunosorbent assay (VT-ELISA). Colonies from the MacConkey agar were inoculated into 500 μl of modified tryptic soy broth (Becton Dickinson) containing 1.5 g/L bile salts No.3, 10 μg/ml vancomycin, and 10 μg/ml cefsulodin (Sigma) (mTSB-VC), and incubated for 18–27 hours at 37°C with shaking at ~150 rpm. One hundred microliters of each resulting culture where then tested in duplicate in the VT ELISA as previously described [25,31]. The absorbance of the tested samples was measured using a SpectraMax M2 Microplate Reader (MTX Lab Systems, Inc., US) at a dual wavelength of 450/620 nm and air as a blank. Samples were considered suspicious or positive for VT if the mean optical density (OD) were 1.5–2.0x or >2.0x that of the negative control, respectively. Suspicious samples were tested a second time. Presumptive VTEC isolates positive by VT-ELISA were inoculated into TSB in preparation for final confirmation using PCR.

Overnight cultures (18–24 h) of presumptive VTEC were confirmed as *E*. *coli* using a monoplex PCR for the *gadA* gene as described by Doumith et al. [32]. They were also tested for both variants of the VT producing gene (i.e., *vt1*, *vt2*) and two other virulence determinants: *eaeA* and *hlyA* by a multiplex PCR previously described by Paton and Paton [33], and modified by the Public Health Agency of Canada *E*. *coli* Reference Lab with the addition primers for detection of *vt2f* (Table 1). The DNA was extracted from lysates using a boiling method previously described by Nadya et al. [25]. Recovered DNA was reserved and stored at -20°C until use. Both monoplex and multiplex PCR reactions were carried out using 2 μl of template DNA, and following the protocol outlined by Nadya et al., with addition of the *vt2f* primers to the multiplex reaction at a concentration of 0.4 μM each.

**Table 1:** Sequences of primers used for confirmation and virulence characterization of verotoxigenic *E*. *coli* isolates.

Gene	Primer	Sequence (5’-3’)	Amplicon Size (bp)	Reference
*gadA*	forward	GATGAAATGGCGTTGGCGCAAG	373	[32]
	reverse	GGCGGAAGTCCCAGACGATATCC		
*vt1*^a^	forward	ATAAATCGCCATTCGTTGACTAC	180	[33]
	reverse	AGAACGCCCACTGAGATCATC		
*vt2*^a^	forward	GGCACTGTCTGAAACTGCTCC	255	[33]
	reverse	TCGCCAGTTATCTGACATTCTG		
*eaeA*	forward	GACCCGGCACAAGCATAAGC	384	[33]
	reverse	CCACCTGCAGCAACAAGAGG		
*hlyA*	forward	GCATCATCAAGCGTACGTTCC	534	[33]
	reverse	AATGAGCCAAGCTGGTTAAGCT		
*vt2f*	forward	GAACAGATGGAATTTGCAGCCA	112	[34]
	reverse	TAAACTTCACCTGGGCAAAGCC		

^a^The primers for the genes *vt*1 and *vt2* are equivalent to *stx*1 and *stx2* as they are sometimes labelled in other papers.

Recovered VTEC isolates were serotyped at the NML Guelph *E*. *coli* Reference Laboratory by accredited serological agglutination methods [35].

### *Listeria monocytogenes* detection, isolation, and characterization

The presence of *L*. *monocytogenes* was detected in water samples by Silliker JR Laboratories (Burnaby, BC) using the MFHPB-30 method from Health Canada [36]. Briefly, 25 ml of sample was enriched in *Listeria* enrichment broth for 24 and 48 hours at 35°C before being subsequently used to inoculate modified Fraser Broth (MFB) and incubated for 24–26 hours at 35°C. Positive enrichments in MFB were then streaked onto Oxford agar and PALCAM agar and incubated for up to 48 hours at 35°C. A minimum of five typical colonies from each plating were then tested for hemolysis, motility, and carbohydrate utilization to confirm the identity as *L*. *monocytogenes*. Positive isolates were then recovered and submitted for serotyping analysis.

*L*. *monocytogenes* isolates were characterized using PFGE with AscI and ApaI restriction endonucleases, as previously described [37] and according to current PulseNet guidelines. The PFGE patterns were analysed using BioNumerics 6.5 (Applied Maths, Belgium). Pattern clustering was performed using the unweighted pair group method (UPGMA) with a DICE correlation of 1.0%. PFGE patterns were compared against the national PulseNet Canada Listeria database to assign designations.

Serotyping of *L*. *monocytogenes* was done using the commercial O-antigen *Listeria* antisera (Denka Seiken, Tokyo, Japan), according to the manufacturer’s recommendations.

### *Salmonella* detection, isolation, and characterization

The presence of *Salmonella* was detected in the water samples by Silliker JR Laboratories (Burnaby, BC) using the MFHPB-20 method from Health Canada [38]. Briefly, 25 ml of sample was non-selectively enriched for 18 to 24 hours at 35°C in buffered peptone water, followed by selective enrichment of these cultures in Tetrathionate Brilliant Green broth and Rappaport-Vassiliadis Soya Peptone broth for 24 ± 2 hours at 42.5°C. Selective enrichment cultures were then streaked onto Bismuth Sulfite agar and Brilliant Green Sulfa agar and incubated for 24 ± 2 hours at 35°C. Suspected colonies were subjected to biochemical screening for carbohydrate utilization, H_2_S production, and gas formation on Triple Sugar Iron agar; presence and absence of lysine decarboxylase and lysine deaminase, respectively, on Lysine Iron agar; and absence of urease on Christensen’s Urea agar. Positive isolates were then recovered and submitted for serotyping analysis.

Recovered *Salmonella* isolates were serotyped at the World Organization for Animal Health (WOAH/OIÉ) Reference Laboratory for Salmonellosis at NML Guelph by accredited serological agglutination methods [35,39].

### Enumeration of total fecal coliforms and generic *E*. *coli*

Generic *E*. *coli* and total fecal coliforms (TFC) were enumerated in each sample using two distinct methods: 1) membrane filtration followed by plating on selective differential media, and 2) 3M™ Petrifilm™ E. coli/Coliform Count Plates.

For the membrane filtration method, a 25 ml aliquot of each water sample was vacuum filtered through an 85 mm, 0.45 μm pore size GN-6 Metricel® membrane filter disc (Pall Laboratory, St. Laurent, PQ), which was subsequently transferred to m-FC agar medium (Hardy Diagnostics, Santa Maria, CA) containing 1% rosolic acid (Hach Canada Ltd., London, ON) and incubated at 44.5 ± 1°C for 18–24 hours [40]. All resulting colonies with a blue colour were counted as fecal coliforms. The filter membranes were then transferred from the m-FC media to nutrient agar medium containing 4-methylumbelliferyl β-D-glucuronide (NA-MUG; Hardy Diagnostics) and incubated at 37°C for 4–6 hours. Colonies producing fluorescence under long wave UV light (365 nm) were assumed to be generic *E*. *coli* [40].

For the 3M™ Petrifilm™ method, a 1 ml aliquot of each water sample was applied to an *E*. *coli*/Coliform Count Petrifilm™ (3M Science, London, ON) following the manufacturer’s directions, and then subsequently incubated at 44.5 ± 1°C for 18–24 hours. All blue and red colonies showing evidence of gas production were assumed to be fecal coliforms, and all gas-producing blue colonies were assumed to be *E*. *coli*.

### Environmental factors associated with the occurrence of pathogens

The temperature of the water samples was measured at the time of sampling using an alcohol thermometer. The thermometer was inserted into the sample within one minute of collection, and allowed to equilibrate for at least 20 seconds before reading. The pH of the samples was measured back at the laboratory within 36 hours after collection, and in duplicate using an Accumet digital pH meter (Fisher Scientific, Ottawa, ON).

Total dissolved solids (TDS) were measured for each sample by oven drying. Briefly, foil weigh boats were dried over night at 120°C before pre-weighing. A 25 ml aliquot of each water sample was added to a pre-dried and pre-weighed weigh boat in duplicate after the water samples were allowed to settle for > 2 hours. The aliquots were dried overnight (~16 hours) at 120°C and allowed to cool at room temperature in a desiccator before determining the final weight of the remaining TDS.

Weather data was collected from the Environment Canada website (http://climate.weather.gc.ca/historical_data/search_historic_data_e.html) for the sampling areas on each sampling date. Data for the Serpentine watershed sampling sites were collected from the Pitt Meadows CS weather station (49°12'29.964" N; 122°41'24.076" W), and data for the Sumas watershed sampling sites were collected from the Sumas Canal weather station (49°06'48.008" N; 122°06'35.004" W) with missing data being filled in from the Mission West Abbey weather station (49°09'09.002" N; 122°16'14.001" W). The weather data collected included total precipitation and average temperature on the date of sampling as well as on each of the five days prior to sampling.

Water flow direction at the sample sites was determined by visual inspection during each sampling. The upstream water sources were determined using the Drainage Mains and Drainage Open Channels datasets collected from the City of Surrey’s Open Data Site (http://data.surrey.ca), the City of Abbotsford Map Viewer (maps.abbotsford.ca), and the City of Chilliwack Webmap (maps.chilliwack.com). Livestock information was collected from the Agricultural Land Use Inventories (ALUI) for Surrey (2010), Abbotsford (2012), and Chilliwack (2012), retrieved from the Government of British Columbia (http://www2.gov.bc.ca/gov/content/industry/agriculture-seafood/agricultural-land-and-environment/strengthening-farming/planning-for-agriculture/agricultural-land-use-inventories/south-coast).

Geographic data was analyzed and map (Fig 1) was created using ArcMap (ArcGIS, version 10.2.2; http://www.arcgis.com). Upstream water sources were determined up to three kilometers from each sample site. Upstream water sources were considered to be any waterway directly connected to the sample site where the water was determined, either from retrieved geographic data or direct observation, to flow to the sample site. If livestock was present on a property on the ALUI, that whole property was considered to be positive for the presence of that livestock type. Livestock was considered to be cow (i.e., dairy or beef), poultry, or other (i.e., swine, sheep, goat). Livestock were considered to be upstream if the property directly bordered on to the connecting waterway. Livestock properties separated from the waterway by a road were not considered to be bordering on the waterway. Using the measurement tool in ArcMap, the upstream distance to the nearest livestock of each type was measured for each livestock type. Also measured was the total length of livestock associated property bordering the upstream waterways up to a distance of 1 km, 2 km, and 3 km. Any lengths of upstream waterway that passed through a livestock property were counted twice to account for bordering on two banks of the waterway.

### Statistical analysis

Statistical analysis of results was conducted using R version 3.2.3 (R Core Team, 2015; http://www.R-project.org). Uniformity of occurrence within and between watersheds was completed using either the χ^2^ test when appropriate, or the Fisher’s Exact test when frequencies of occurrence less than five were present in the calculations. Correlations between pathogen occurrence and indicator concentrations, weather, and water characteristics were calculated using the point-biserial method, which correlates a continuous variable with a dichotomous variable [22], using the ltm software package (http://www.jstatsoft.org/v17/io5/). Fisher’s LSD tests were conducted using the agricolae software package (version 1.2–4; https://CRAN.R-projects.org/package=agricolae).

## Results

### Pathogen occurrence

A total of 223 samples were collected over the course of this study, with an overall pathogen occurrence of 15.7%. The occurrence of VTEC, *L*. *monocytogenes*, and *Salmonella* are summarized in Table 2. *L*. *monocytogenes* was the most commonly recovered pathogen at 10.3%, followed by VTEC and *Salmonella* with 4.9% and 2.7% occurrence, respectively.

**Table 2:** Occurrence of verotoxigenic *E*. *coli* (VTEC), *L*. *monocytogenes*, and *Salmonella* in irrigation water collected from seven sites in the Sumas and Serpentine watersheds.

Site	# of Samples	Number of positive samples (% of positive samples)			
		VTEC	*L*. *monocytogenes*	*Salmonella*	Any Pathogen
Sumas 1a	35	4 (11.4%)	1 (2.86%)	3 (8.57%)	6 (17.1%)
Sumas 1b	26	0	0	2 (7.69%)	2 (7.69%)
Sumas 2	34	0	1 (2.94%)	0	1 (2.94%)
Sumas 3	34	1 (2.94%)	4 (11.8%)	0	5 (14.7%)
Serpentine 1	30	0	4 (13.3%)	1 (11.1%)	4 (13.3%)
Serpentine 2	34	5 (14.7%)	9 (26.5%)	0	12 (35.3%)
Serpentine 3	30	1 (3.33%)	4 (13.3%)	0	5 (16.7%)
**Total Sumas**	129	5 (3.88%)*	6 (4.65%)^A^	5 (3.88%)*	14 (10.9%)^A^
**Total Serpentine**	94	6 (6.38%)*	17 (18.1%)^B^	1 (1.06%)	21 (22.3%)^B^
**Total**	223	11 (4.93%)	23 (10.3%)	6 (2.69%)	35 (15.7%)

^A,B^ Pathogens at watersheds with different letters represent a significant difference in pathogen occurrence between the two watersheds (χ^2^; p < 0.05).

* A significant difference in pathogen occurrence between sites within this watershed was observed (Fisher’s exact test; p < 0.10).

The recovery of pathogens was more common in the Serpentine watershed compared to the Sumas watershed, with 22.3% compared to 10.9% occurrence, respectively (χ^2^; p = 0.032). This difference was primarily due to the increased occurrence of *L*. *monocytogenes* observed in the Serpentine watershed compared to the Sumas watershed: 18.1% and 4.65%, respectively (χ^2^; p = 0.002). Neither VTEC nor *Salmonella* showed evidence for differences in occurrence between the two watersheds (χ^2^; p > 0.05), although all but one occurrence of *Salmonella* was derived from the Sumas watershed. Site specific differences were observed for VTEC occurrence within both the Serpentine watershed (Fisher’s exact test; p = 0.046) and the Sumas watershed (Fisher’s exact test; p = 0.075), with all but one positive sample in each watershed coming from a single site. Moreover, VTEC was not recovered at one and two sites in the Serpentine and Sumas watersheds, respectively. Similarly, *Salmonella* showed evidence of site-specific occurrence in the Sumas watershed (Fisher’s exact test; p = 0.097). No significant site-specific differences were observed for *L*. *monocytogenes* in either watershed, though two thirds of positive samples from the Sumas watershed came from a single site.

To investigate the effects of seasonal changes on pathogen occurrence, relative occurrence was investigated for winter (December to February), spring (March to May), summer (June to August), and fall (September to November) months. These results are summarized in Table 3. The recovery of any pathogen was more common during the fall and winter with 28.6% and 23.3% positive samples, respectively, compared to 15.6% for spring and 6.2% for summer (χ^2^; p = 0.006). This was likely driven by differences in recovery of *L*. *monocytogenes* which showed 22.9% occurrence in the fall and 16.3% occurrence in the winter, compared to 7.8% and 3.7% for spring and summer, respectively (Fisher exact test; p = 0.007). No significant difference in occurrence of VTEC or *Salmonella* was observed, though the lowest occurrence for both pathogens was observed to be during the summer.

**Table 3:** Seasonal occurrence of verotoxigenic *E*. *coli* (VTEC), *L*. *monocytogenes*, *Salmonella*, or any of the three in the Sumas and/or Serpentine watersheds.

	Winter	Spring	Summer	Fall
**Sumas Watershed**				
VTEC	2/25 (8%)	2/39 (5.1%)	0/45 (0%)	1/20 (5%)
*L*. *monocytogenes*	2/25 (8%)	1/39 (2.6%)	2/45 (4.4%)	1/20 (5%)
*Salmonella*	2/25 (8%)	1/39 (2.6%)	1/45 (2.2%)	1/20 (5%)
Any	5/25 (20%)	4/39 (10.3%)	3/45 (6.7%)	2/20 (10%)
**Serpentine Watershed**				
VTEC	1/18 (5.6%)	3/25 (12%)	1/36 (2.8%)	1/15 (6.7%)
*L*. *monocytogenes*^α^	5/18 (27.8%)	4/25 (16%)	1/36 (2.8%)	7/15 (46.7%)
*Salmonella*	0/18 (0%)	0/25 (0%)	0/36 (0%)	1/15 (6.7%)
Any^α^	5/18 (27.8%)	6/25 (24%)	2/36 (5.6%)	8/15 (53.3%)
**Both Watersheds**				
VTEC	3/43 (7%)	5/64 (7.8%)	1/81 (1.2%)	2/35 (5.7%)
*L*. *monocytogenes*^α^	7/43 (16.3%)	5/64 (7.8%)	3/81 (3.7%)	8/35 (22.9%)
*Salmonella*	2/43 (4.7%)	1/64 (1.6%)	1/81 (1.2%)	2/35 (5.7%)
Any^α^	10/43 (23.3%)	10/64 (15.6%)	5/81 (6.2%)	10/35 (28.6%)

Winter = Dec-Feb; Spring = Mar-May; Summer = Jun-Aug; Fall = Sep-Nov

^α^ Significant seasonal variation was observed for this pathogen(s) within respective watershed(s) (Fisher exact test; p < 0.05).

Significant seasonal differences were observed for the Serpentine watershed, but not for the Sumas watershed. These differences echoed those of both watersheds together, with significant differences being observed for *L*. *monocytogenes* (Fisher exact test; p = 0.0008) or any of the three pathogens (Fisher exact test; p = 0.002), but not for VTEC or *Salmonella*. The occurrence of any of the three pathogens in the Serpentine watershed was highest during the fall with 53.3% occurrence, compared to 27.8%, 24%, and 5.6% for winter, spring, and summer, respectively. Similarly, the occurrence of *L*. *monocytogenes* was greater in the fall at 46.7% occurrence compared to 27.8% occurrence in the winter, 16% occurrence in the spring, and only 2.8% occurrence in the summer.

### Pathogen characteristics

Of the 11 samples positive for VTEC, eight distinct VTEC serotypes were observed, though isolates from two positive samples were not obtained for serotyping. These results are summarized in Table 4. There was no occurrence of the same serotype being observed at different sites; however, VTEC O69:H11 was observed on two consecutive sampling dates at Serpentine 2. Also of note was the recovery of O103:H2, a member of the “Big Six” non-O157 VTEC associated with human disease.

**Table 4:** Serotype and virulence genes of verotoxigenic *E*. *coli* isolates collected from irrigation water at three sites each in the Sumas and Serpentine watersheds of the Lower Mainland of British Columbia.

Serotype	Site	Month collected	*vt1*	*vt2*	*eaeA*	*hlyA*
O103:H2	Sumas 1	Feb 2015	+	-	+	+
O109:H5	Sumas 1	Mar 2015	+	-	-	-
O116:H25	Serpentine 2	May 2015	-	+	+	+
Unknown 1^a^	Serpentine 2	Sep 2015	-	+	+	+
O153:NM	Sumas 1	Nov 2015	-	+	+	-
O76:H19	Sumas 1	Feb 2016	+	-	-	+
O69:H11	Serpentine 2 Serpentine 2	Feb 2016 Mar 2016	+	-	+	+
Unknown 2^a^	Serpentine 2	Apr 2016	-	+	+	+
O34:H2	Sumas 3	May 2016	-	+	+	-
O22:H8	Serpentine 3	May 2016	+	-	-	+

^a^These strains were not isolated for serotyping.

In order to further characterize the recovered VTEC isolates, the presence of four genes associated with virulence were investigated: the toxin producing genes *vt1* and *vt2*; the *eaeA* gene, encoding intimin, responsible for intimate host-cell attachment; and *hlyA*, producing the pore-forming hemolysin toxin. The results are summarized in Table 4. There was an equal occurrence of both toxin genes, and no recovered isolate showed the presence of both toxin genes. The virulence genes *eaeA* and *hlyA* were each present in eight of the eleven VTEC isolates recovered, with six isolates showing the presence of both.

Three distinct serotypes of *L*. *monocytogenes* were observed over the course of this study and are summarized in Table 5. The serotype 1/2a was the most common (15 occurrences) followed by 4b (10 occurrences), 1/2b (2 occurrences), and 4c (1 occurrence). Within the Serpentine watershed, serotype 1/2a and 4b were equally common with 10 recoveries each, along with one recovery each of 4c and 1/2b. This is in contrast with the Sumas watershed, where only serotype 1/2a was ever recovered except for one occurrence of 1/2b. Also of note is that on five separate sampling dates, two different serotypes were observed at the same site. This occurred four times for sample site Serpentine 2, and once for Serpentine 1.

**Table 5:** Listeria serotypes collected from irrigation water at three sites each in the Sumas and Serpentine watersheds of the Lower Mainland of British Columbia.

Serotype	Sumas Watershed		Serpentine Watershed	
	Recoveries	Sites	Recoveries*	Sites
1/2a	5	2,3	11	1,2,3
1/2b	1	1a	1	2
4b	0	-	10	1,2,3
4c	0	-	1	1

*Occasional recoveries of multiple serotypes were observed at the same site on the same date in the Serpentine watershed.

Nineteen unique PFGE fingerprints were observed among *L*. *monocytogenes* isolates recovered from a total of 25 water samples (S1 Table). Three sample sites showed recurring PFGE fingerprints on multiple sampling dates. The same PFGE fingerprint was observed twice at Serpentine 1, four times at Serpentine 2, (the same PFGE fingerprint all four times), and twice at Sumas 3. Furthermore, two PFGE fingerprints were observed at two different sampling sites within the Serpentine watershed: Serpentine 1 and Serpentine 2. Of particular interest, one of these multi-site PFGE fingerprints was also a recurring fingerprint, being recovered twice at Serpentine 1, and four times at Serpentine 2.

Of the six *Salmonella* isolates recovered, four different serotypes were observed, summarized in Table 6. The most common, and only recurring serotype was *S*. Enteritidis, which was recovered three separate times. Interestingly, these recoveries were at two different sites; however, these two sites are in close proximity and share an upstream source, so it is likely that this may have come from a single location.

**Table 6:** *Salmonella* serotypes collected from irrigation water at three sites each in the Sumas and Serpentine watersheds of the Lower Mainland of British Columbia.

Serotype	Sumas Watershed		Serpentine Watershed	
	Recoveries	Sites	Recoveries	Sites
Typimurium	1	1a	0	-
Enteritidis	3	1a, 1b	0	-
Daytona	0	-	1	1
Heidelberg	1	1a	0	-

### Indicator organisms

In order to determine if TFC or generic *E*. *coli* concentrations are reliable indicators of pathogen presence, point-biserial correlations (r_pb_) were calculated for each indicator/method combination with respect to each pathogen. These correlations are summarized in Table 7. The occurrence of any of the three pathogens was significantly correlated with both TFC (r_pb_ = 0.448; n = 190; p < 0.001), and generic *E*. *coli* (r_pb_ = 0.430; n = 176; p < 0.001) when measured using the membrane filtration method, but only with TFC (r_pb_ = 0.252; n = 221; p < 0.001) when measured using the Petrifilm™ method; moreover, the correlation coefficient was much lower for the Petrifilm™ method.

**Table 7:** Point-biserial correlation coefficients (r_pb_) of total fecal coliforms and generic *Escherichia coli* with the occurrence of verotoxigenic *E*. *coli* (VTEC), *Salmonella*, and *Listeria monocytogenes* in water samples collected from three sites each in two watersheds of the Lower Mainland of British Columbia.

	Total Fecal Coliforms		Generic *E*. *coli*	
Pathogen	Membrane Filtration	3M™ Petrifilm™	Membrane Filtration	3M™ Petrifilm™
VTEC	0.273**	0.267**	0.320**	0.092
*L*. *monocytogenes*	0.412**	0.152*	0.403**	0.072
*Salmonella*	0.097	0.042	0.099	-0.017
Any pathogen	0.448**	0.252**	0.430**	0.083

* p < 0.05

** p < 0.001

For individual pathogens, TFC were significantly correlated with the occurrence of VTEC using both the membrane filtration method (r_pb_ = 0.273; n = 190; p < 0.001) and by the Petrifilm™ method (r_pb_ = 0.267; n = 221; p < 0.001). Generic *E*. *coli* were only significantly correlated when measured using the membrane filtration method (r_pb_ = 0.320; n = 176; p < 0.001). The same pattern was observed for *L*. *monocytogenes* where the occurrence was significantly correlated with TFC by both the membrane filtration method (r_pb_ = 0.412; n = 190; p < 0.001) and by Petrifilm™ (r_pb_ = 0.152; n = 221; p = 0.024), but only significantly correlated with generic *E*. *coli* by the membrane filtration method (r_pb_ = 0.403; n = 176; p < 0.001). No significant correlations were observed for either indicator by either method for the occurrence of *Salmonella*.

In order to investigate how pathogen occurrence associated with changes in indicator concentrations at individual sites, the mean and maximum observed concentrations for TFC and generic *E*. *coli* measured by the membrane filtration method were determined and are shown in Table 8. The concentrations of both indicators were significantly greater in the Serpentine watershed than in the Sumas watershed (Wilcoxon rank sum test; p < 0.001). Similarly, within each watershed, site-specific differences in indicators concentrations were also observed (Kruskal-Wallace test; p < 0.05). Pathogen occurrence was associated with higher mean concentrations of indicators (Wilcoxon rank sum test; p < 0.001); however, as shown in Table 9, only about half of pathogen occurrences were associated with indicator concentrations above the mean for their respective sampling sites.

**Table 8:** Average total fecal coliform and generic *E*. *coli* concentrations for sites sampled during this experiment.

Sample Site	Mean F. coliforms (CFU/100ml)	Max. F. coliforms (CFU/100ml)	Mean Generic *E*. *coli* (CFU/100ml)	Max. Generic *E*. *coli* (CFU/100ml)
Sumas 1a	185 ± 173	806	111 ± 116	502
Sumas 1b	135 ± 144	544	79 ± 95	388
Sumas 2	49 ± 62	246	30 ± 41	154
Sumas 3	85 ± 123	396	42 ± 58	208
Serpentine 1	239 ± 271	1108	136 ± 157	630
Serpentine 2	267 ± 300	970	174 ± 213	700
Serpentine 3	393 ± 281	926	251 ± 197	720

**Table 9:** Ratio of pathogen recoveries associated with indicator organism levels above the mean or the mean plus standard deviation for the respective sampling site.

	Total fecal coliforms		Total generic *E*. *coli*	
Pathogen	> Mean	> Mean + SD	> Mean	> Mean + SD
VTEC	54.5%	36.4%	54.5%	36.4%
*L*. *monocytogenes*	56.5%	39.1%	56.5%	34.8%
*Salmonella*	50.0%	16.7%	50.0%	16.7%
Any pathogen	48.6%	34.3%	48.6%	28.8%

### Environmental factors associated with the occurrence of pathogens

Water temperature, pH, and TDS were measured for each site and are summarized in Table 10. No significant differences were observed in the temperature of the water between sites over the course of sampling, but a significantly lower pH was observed in the Serpentine watershed compared to the Sumas watershed, specifically in Serpentine 2 (Fisher’s LSD; p < 0.05). In addition, the mean pH among all sites was alkaline (i.e., 7.1–7.7), but acidic pH (i.e., 6.5–7.0) was occasionally observed, specifically at Serpentine 2 where a pH of 3.8 and 5.6 was also observed. A greater amount of TDS was also observed at Serpentine 2 compared to the other five sites tested (Fisher’s LSD; p < 0.05).

**Table 10:** Water temperature, pH, and total dissolved solids (TDS) observed at the six primary sampling sites monitored during this study.

Site	Temperature (°C)	pH*	TDS (mg/ml)*
Sumas 1	14.3 ± 5.4	7.64 ± 0.32^a^	0.254 ± 0.224^a^
Sumas 2	15.3 ± 5.8	7.59 ± 0.28^a^	0.212 ± 0.097^a^
Sumas 3	15.5 ± 6.4	7.72 ± 0.30^a^	0.276 ± 0.126^a^
Serpentine 1	12.7 ± 4.4	7.35 ± 0.43^b^	0.205 ± 0.095^a^
Serpentine 2	14.5 ± 5.5	7.11 ± 0.83^c^	0.382 ± 0.232^b^
Serpentine 3	13.9 ± 4.8	7.56 ± 0.33^ab^	0.219 ± 0.283^a^

*Values in the same column with different superscripts indicated a significant difference (Fisher’s LSD; p < 0.05).

Correlations between pathogen occurrence and (i) water characteristics, (ii) levels of precipitation and (iii) mean temperature were measured using the point-biserial correlation method; the resulting correlation coefficients are shown in Table 11. Water temperature significantly correlated negatively with pathogen occurrence (r_pb_ = -0.225; n = 216; p < 0.001), as did the pH of the water samples (r_pb_ = -0.279; n = 214; p < 0.001), implying that pathogen occurrence was greater when the water was cooler and closer to a neutral pH. Neither of these correlations is particularly strong, however. No correlation was observed between the total dissolved solids and pathogen occurrence. In relation to correlations with individual pathogens, both water temperature and pH only correlated significantly with the occurrence of *L*. *monocytogenes* (r_pb_ = -0.226; n = 216; p = 0.002 and r_pb_ = -0.326; n = 214; p < 0.001, respectively).

**Table 11:** Point-biserial correlations (r_pb_) of the occurrence of verotoxigenic *E*. *coli* (VTEC), *Salmonella*, and *L*. *monocytogenes* in the Sumas and Serpentine watersheds with physicochemical characteristics of the water (temperature, pH, and total dissolved solids); the levels of precipitation five days prior (P5), four days prior (P4), three days prior (P3), two days prior (P2), one day prior (P1), the day of (P0), and the total volume over the three days prior (P1-3) and five days prior (P1-5) to sample collection; and the average temperature five days prior (T5), four days prior (T4), three days prior (T3), two days prior (T2), one day prior (T1), the day of (T0), and the average temperature over the three days prior (T_avg_1-3) and five days prior (T_avg_1-5) to sample collection.

	VTEC	*L*. *monocytogenes*	*Salmonella*	Any pathogen
**Physicochemical Characteristics**				
Water temperature	-0.115	-0.226***	-0.059	-0.225***
pH	-0.104	-0.326***	-0.005	-0.279***
Total dissolved solids	-0.020	0.031	-0.080	0.008
**Total Precipitation**				
P5	0.128	0.185**	0.135*	0.206**
P4	0.078	0.129	0.135*	0.144*
P3	0.093	0.156*	0.169*	0.189**
P2	0.019	0.074	0.116	0.099
P1	0.199**	0.165*	0.076	0.218**
P0	-0.030	0.037	-0.041	-0.007
P1-3	0.128	0.160*	0.145*	0.207**
P1-5	0.146*	0.195**	0.173*	0.239***
**Average Daily Temperature**				
T5	-0.057	-0.125	0.006	-0.100
T4	-0.022	-0.122	-0.011	-0.089
T3	-0.030	-0.132	-0.015	-0.093
T2	-0.057	-0.138*	-0.012	-0.123
T1	-0.073	-0.171*	-0.032	-0.167*
T0	-0.081	-0.199**	-0.062	-0.189**
T_avg_1-3	-0.054	-0.149*	-0.020	-0.130
T_avg_1-5	-0.050	-0.145*	-0.013	-0.120

* p < 0.05

** p < 0.01

*** p < 0.001

Pathogen occurrence was most strongly correlated with higher levels of total precipitation over the 5 days before sampling (r_pb_ = 0.239; n = 223; p < 0.001), and specifically on the day just before sampling (r_pb_ = 0.218; n = 223; p = 0.001). Significant, but less strong correlations were also observed for precipitation levels three (r_pb_ = 0.189; n = 219; p = 0.005), four (r_pb_ = 0.144; n = 220; p = 0.033), and five (r_pb_ = 0.206; n = 223; p = 0.002) days prior to sampling, and the total amount of precipitation over the three days prior to sample collection (r_pb_ = 0.207; n = 219; p = 0.005). With respect to individual pathogens, VTEC recovery was most strongly correlated with precipitation on the day prior to sampling (r_pb_ = 0.199; n = 223; p = 0.003), and slightly less so with total precipitation over the five days prior to sampling (r_pb_ = 0.146; n = 216; p = 0.032). On the other hand, the recovery of *L*. *monocytogenes* was most strongly correlated with total precipitation over the five days prior to sampling (r_pb_ = 0.195; n = 216; p = 0.004), specifically the single day precipitation five days before sampling (r_pb_ = 0.185; n = 223; p = 0.006), and slightly less correlated with precipitation one day prior to sampling (r_pb_ = 0.165; n = 223; p = 0.014). *Salmonella* occurrence was most strongly correlated with total precipitation over the five days prior to sample collection (r_pb_ = 0.173; n = 216; p = 0.011), and specifically the single day precipitation three days prior to sample collection (r_pb_ = 0.169; n = 219; p = 0.012).

Pathogen occurrence significantly correlated negatively with the mean air temperature on the day of sampling (r_pb_ = -0.189; n = 217; p = 0.005), implying increased pathogen prevalence on cooler days. With respect to individual pathogens, only *L*. *monocytogenes* was correlated with air temperature, primarily on the day of sample collection (r_pb_ = -0.199; n = 217; p = 0.003).

Correlations between pathogen occurrence and nearby upstream livestock were calculated using the Spearman’s rank correlation test (r_s_) and the results are summarized in Table 12. Overall, pathogen occurrence did not significantly correlate with proximity to or density of upstream livestock. Looking at each pathogen individually, however, the occurrence of VTEC correlated significantly with the length of upstream water way that bordered on cow or poultry farms to a distance of 2 km (r_s_ = 0.812; n = 6; p = 0.050) and 3 km (r_s_ = 0.812; n = 6; p = 0.050); although no significant correlation was observed at the 1 km range. For *L*. *monocytogenes*, the likelihood of occurrence was significantly correlated with density of cow farms within 2 km upstream (r_s_ = 0.841; n = 6; p = 0.036). No significant correlations were observed for the likelihood of *Salmonella* occurrence livestock proximity or density.

**Table 12:** Spearman rank correlation coefficients (r_s_) for the occurrence of verotoxigenic *E*. *coli* (VTEC), *Salmonella*, and *L*. *monocytogenes* with proximity and density of upstream livestock.

	VTEC	*L*. *monocytogenes*	*Salmonella*	Any Pathogen
**Nearest Livestock**^A^				
Cow^B^	-0.377	-0.638	-0.135	-0.486
Poultry^C^	-0.464	0.232	-0.507	-0.429
Cow or Poultry	-0.696	-0.319	-0.439	-0.771
Any^D^	-0.493	-0.174	-0.034	-0.543
**Livestock Border (1 Km)**^E^				
Cow	0.257	0.772	0.020	0.338
Poultry	0.399	-0.664	0.775	0.393
Cow or Poultry	0.431	0.493	0.395	0.516
Any	0.397	0.485	-0.034	0.464
**Livestock Border (2 Km)**				
Cow	0.609	0.841*	-0.439	0.657
Poultry	0.308	-0.585	0.359	0.213
Cow or Poultry	0.812*	0.638	-0.372	0.829
Any	0.812*	0.638	-0.372	0.829
**Livestock Border (3 Km)**				
Cow	0.377	0.290	-0.778	0.314
Poultry	0.585	-0.339	0.359	0.516
Cow or Poultry	0.812*	0.203	-0.304	0.771
Any	0.638	0.029	-0.304	0.600

^A^Nearest distance for surface water to travel.

^B^Includes both dairy and beef cattle.

^C^Includes chicken and turkey.

^D^Includes bovine and poultry, as well as swine, sheep, or goats.

^E^Total length of waterway bordered by properties containing livestock within a given distance.

* p < 0.05

## Discussion

### Occurrence, prevalence, and spatial/ temporal spread of pathogens in water

The most commonly observed pathogen was *L*. *monocytogenes*, which was expected due to its relative ubiquity in the natural environment [41], followed by VTEC then *Salmonella*. This trend differs from two previous studies which looked at the occurrence of these three specific pathogens in surface waters from agricultural areas: one from New York State [29], and one from California [30]. Strawn et al. [29] found *L*. *monocytogenes* to be more prevalent than the other two pathogens, but a higher occurrence of *Salmonella* compared to VTEC was also reported. Similarly, Cooley et al. [30] also reported *Salmonella* to occur more often than VTEC, but also more often than *L*. *monocytogenes*. The reduced representation of VTEC in these two studies can potentially be explained by differences in detection methods. The VT-IB method used in this study has been shown to increase VTEC recovery from 7.5% to 32% when compared to methods involving a pre-enrichment such as those used by Strawn et al. [29] and Cooley et al. [30].

The overall recovery rate of VTEC during the course of this study was 4.93%; however, the recovery rate ranged from 3.9% to 6.4% between watersheds, and three sites showed no occurrence of VTEC. These site-specific trends are consistent with previous studies [25,30,31] and imply that there is a geographical dependence on VTEC occurrence. These differences may be attributed to proximity to a host source, such as livestock, or the application of manure to neighbouring fields, as pathogen occurrence has been previously observed to be higher in agricultural areas compared to areas unaffected by farming [19]. Indeed, in the current study, the occurrence of VTEC was observed to correlate with increased density of upstream livestock.

The overall occurrence for *L*. *monocytogenes* in this study was 11.2%, but differed significantly between the two watersheds, ranging from 4.7% to 20.2%. This may imply geographical or environmental effects on *L*. *monocytogenes* occurrence such as has previously been observed for *L*. *monocytogenes* occurrence in soil [41]. Indeed, the correlation observed with upstream livestock in this study may imply a potential source for the contamination as domestic cattle are known to be a common carrier of *L*. *monocytogenes* [42,43], and previous studies have found the occurrence of *L*. *monocytogenes* to correlate with proximity to pasture [21] and upstream dairy farms [20]. *L*. *monocytogenes* occurrence was also significantly higher during the Fall and Winter, which is similar to California where occurrence was highest during the Winter and Spring [30]. Temperature may be a driving force of this phenomenon, as survival of *L*. *monocytogenes* in water has been shown to double at 4°C when compared to 20°C [44] implying longer persistence of contaminating pathogens.

The overall recovery of *Salmonella* across all sites during this study was 2.69%, but ranged from 7.7% to 11.1% across the three sites where the pathogen was recovered. No significant difference in occurrence for *Salmonella* was observed between watersheds, but some evidence of site-dependence was observed in the Sumas watershed. It should also be noted that the two sites in the Sumas watershed where *Salmonella* was isolated were in close proximity and share a common source. Previous studies have also shown differences in *Salmonella* occurrence across various sampling sites [30], and have suggested proximity to agriculture to increase occurrence [19]. No seasonal variation in *Salmonella* occurrence was observed despite being reported in previous studies [27,30,45], but may be the result of the low recovery rate for the pathogen in this study.

### Pathogen characteristics

Of the eight distinct VTEC serotypes observed during this study, no occurrence of VTEC O157:H7 was observed, and only one, O103:H2, is a member of the “Big 6” non-O157 serotypes associated with a high proportion of severe human illness [46]. VTEC O103:H2 has been isolated from human cases of illness both in North America [47], and Europe [48,49], and was responsible for an outbreak at a nursery school in Japan [50]. Furthermore, serotype O103 is commonly associated with illness in British Columbia [51]. The absence of VTEC O157 recovery was interesting since every other recent survey of water in Canada has shown the presence of VTEC O157:H7, although always at low levels between 1% and 3% [19,27,45,52]. Other disease associated serotypes observed during this study included O76:H19 which was responsible for a household outbreak of bloody diarrhea in Spain [53], and O22:H8 which has been associated with bloody diarrhea in Germany [54] and isolated from patients exhibiting symptoms of HUS [55]. Members of serogroups O69 and O153 have also been isolated with patients exhibiting diarrhea in India [56].

Genotypic analysis of VTEC showed an even proportion of isolates possessing *vt1* and *vt2*, with no recovered isolates possessing both toxin genes. A previous study in the same area, but with a higher VTEC recovery, however, found *vt1* to be more common that *vt2*, with 10% of recovered isolates possessing both variants of the toxin gene [25]. The intimin gene, *eaeA*, was observed in seven of the nine serotypes, and was more commonly associated with *vt2*, which is of concern since the presence of these two genes has been most associated with EHEC symptoms, (*i*.*e*., hemorrhagic colitis, hemolytic-uremic syndrome) in humans [57]. Observed serotypes with *vt2* and *eaeA* included O116:H25, O153:NM, O34:H2, and both of the un-serotyped VTEC isolates. On the other hand, *hlyA* was also present in seven of the observed serotypes, but was more commonly associated with *vt1* which is similar to observations by Nadya et al. There have been few studies of virulence factors associated with VTEC isolated from surface waters, but a recent survey of VTEC isolated from produce by the United States Food and Drug Administration found that *vt2* was over five times more common than *vt1* in those isolates, with the presence of *eaeA* and *hlyA* being 9% and 61%, respectively [58].

Over the course of this study, the recovered isolates of *L*. *monocytogenes* were overwhelmingly members of the serotypes 1/2a and 4b except for one isolate deemed to be serotype 4c and one isolate 1/2b. This is of concern since the serotypes 1/2a, 1/2b, and 4b are responsible for the majority of human listeriosis cases [59], including large outbreaks associate with cantaloupes [60], caramel apples [61] and packaged salads [62]. To the best of the authors’ knowledge, no outbreaks associated with serotype 4c have been reported.

Persistence of certain strains was also observed using PFGE analysis of the recovered *L*. *monocytogenes* isolates. Multiple recoveries of isolates showing the same PFGE pattern were observed at three sites, suggesting persistent upstream point sources. More importantly, however, isolates of serotype 4b showing a single identical PFGE pattern were recovered four times from Serpentine 2 and once from Serpentine 1 over an 11-month period. One other PFGE fingerprint relating to serotype 4b was also shared between Serpentine 1 and Serpentine 2, but only occurred once at each site. It could be speculated that that wild animals may be a reservoir for transporting *L*. *monocytogenes* within the Serpentine watershed, as serotype 4b has been associated with wild animals [63]. Whole genome sequencing of these isolates in the future may provide more insight into their genetic relatedness.

Over the course of this study, four different *Salmonella* serotypes were recovered, including Enteritidis, Typhimurium, and Heidelberg; the most commonly observed serotypes associated with disease in Canada [64]. The only one recovered more than once was *S*. Enteritidis, which was observed at two sites in the Sumas watershed. *S*. Enteritidis has been implicated previously in outbreaks related to sprouts [65,66], and accounted for approximately 50% of infections observed in British Columbia in 2014 and 2015 [67]. *S*. Typhimurium has been implicated in outbreaks involving tomatoes [68] and a recent outbreak associated with cantaloupe [65].

### Indicator organisms

The detection of foodborne pathogens in irrigation water is both time consuming and costly. Therefore, the quality of irrigation water in many parts around the world is primarily assessed using hygiene indicators such as fecal coliforms and generic *E*. *coli* [69]. Significant debate currently exists, however, on whether or not measurements based on these organisms truly predict the risk of foodborne pathogens, with a variety of studies showing both positive and negative correlations [17].

Relative to the ability of each method to predict the occurrence of any of the three pathogens used in this study, the membrane filtration method showed a greater correlation when compared to the Petrifilm™ method. Further, any significant correlations observed using the Petrifilm™ method were also evident with the membrane filtration method, and in all cases, the membrane filtration method produced a higher correlation coefficient. These differences may be due to differences in sample volumes used for enumeration, but may also be the result of differences selective agents in the media. For the remainder of this discussion, references to TFC and generic *E*. *coli* enumerations will be in relation to the membrane filtration method.

Total fecal coliforms showed a marginally higher correlation with total pathogen occurrence than generic *E*. *coli*; although this was not necessarily the case for the occurrence of specific pathogens. Generic *E*. *coli* was a better predictor for the occurrence of VTEC, while *L*. *monocytogenes* correlated slightly better with TFC. Neither indicator was significantly correlated with the presence of *Salmonella*, but this may be the result of the overall lower occurrence of the bacterium, primarily being recovered at only one sampling site.

Despite the significant correlation between pathogen occurrence and indicator concentrations observed in this study, it is questionable as to how powerful these indicators are in predicting the presence of pathogens. TFC and generic *E*. *coli* concentrations were higher, on average, in samples positive for the presence of a pathogen than samples where pathogens were not recovered (Wilcoxon rank sum test; p < 0.0001), but often times, however, these pathogens also occurred when the indicators were at low levels. Indeed, approximately half of pathogen occurrences were observed when indicator concentrations were below the mean concentration for that site. This implies that higher overall risk may be associated with sites showing higher average indicator concentrations, but little prediction can be made from the indicator concentration of a single sample. This lends strength to the FSMA regulations for water quality, which are based on the geometric mean of 20 samples as opposed to a single value [16].

### Environmental factors

The occurrence of any of the three pathogens was significantly, but weakly correlated with lower temperatures at the time of sampling, specifically the occurrence of *L*. *monocytogenes*. It is difficult to say whether or not this observation is related to seasonal or precipitation effects, since precipitation was also correlated with pathogen occurrence, and the Lower Mainland is known for significant rainfall during the winter months. Correlation with precipitation is consistent with previous studies [22–26]. The effect of rainfall is suggested to increase the transport of pathogens into surface waters [70], and carry them longer distances downstream [71]; though it has been suggested that pathogens surviving in sediment reservoirs may also be released due to heavy rainfall [71]. On the other hand, the observation that rain as far back as five days before sampling also correlates with pathogen occurrence may indicate that the high level of precipitation associated with the cooler months in the Lower Mainland may be confounding with seasonal variation in pathogen occurrences.

Another potential factor involved in the seasonal variability is the change in waterflow. The reduced precipitation may inhibit the ability of pathogens to travel down stream, but the region also dams the irrigation ditches during the growing season to ensure sufficient water availability for irrigation. This damming of the ditches results in stagnant water during the summer months compared to the free-flowing streams observed during the winter. It is highly probable that the low pathogen isolation rate during the summer months is attributed to this simple change in water flow.

Water pH was also found to significantly correlate negatively with overall pathogen occurrence, specifically with *L*. *monocytogenes*. The mean pH across sampling sites ranged from 7.11 to 7.72, but Serpentine 2 was singular in having a significantly lower mean pH than all of the other sample sites (Fisher’s LSD; p < 0.05), and also had the highest recovery of *L*. *monocytogenes*. Since the spread of pH was so small, it is likely that this observed relationship is merely a coincidence.

## Conclusion

VTEC, *L*. *monocytogenes*, and *Salmonella* are present in the surface waters used for irrigation in the Lower Mainland of British Columbia, with *L*. *monocytogenes* being the most common, followed by VTEC then *Salmonella*. Of concern is that serotypes commonly associated with human illnesses in British Columbia were recovered for all three pathogens. The frequency of occurrence of these pathogens correlated with higher average concentrations of TFC and generic *E*. *coli* at the site, suggesting that higher mean levels of indicators organisms indeed predict a higher risk for the presence of foodborne pathogens over the long run, but a single sample cannot predict the presence of foodborne pathogens at that particular point in time. Other potential predictors of pathogen presence may include proximity to livestock, cooler weather/seasons, and increased precipitation. These potential predictors, however, are difficult to separate from each other, especially with the low frequency of pathogen recovery in this study. Hypothesis driven experiments of each factor should be conducted in the future to determine their individual effects on pathogen occurrence.

## Supporting information

S1 Table Serotype and PFGE patterns of *L*. *monocytogenes* isolates collected during this study. (DOCX)
