# Supplementary material for: Occurrence, characterization, and potential predictors of verotoxigenic Escherichia coli, Listeria monocytogenes, and Salmonella in surface water used for produce irrigation in the Lower Mainland of British Columbia, Canada
Source: PLoS One. 2017 Sep 27;12(9):e0185437. doi: 10.1371/journal.pone.0185437 (PMC5617201; doi:10.1371/journal.pone.0185437)
Supplement: S1 Table — (DOCX) [file pone.0185437.s001.docx]

S1 Table. Serotype and PFGE patterns of *L. monocytogenes* isolates collected during this study.

| **Serotype** | **PFGE-AscI** | **PFGE-ApaI** | **Site** | **Date** |
| --- | --- | --- | --- | --- |
| 1/2a | LMACI.0172 (03-7760) | LMAAI.0217 | Serpentine 2 | Mar, 2015 |
|  | LMACI.0906 | LMAAI.0909 (PNC_9089) | Sumas 3 | Mar, 2015 |
|  | LMACI.0084 (BOM_4) | LMAAI.0531 | Sumas 3 | Jun, 2015 |
|  | LMACI.0216 | LMAAI.1338 | Serpentine 1 | Sep, 2015 |
|  | LMACI.0041 | LMAAI.0033 | Serpentine 3 | Sep, 2015 |
|  | LMACI.0011 | LMAAI.0015 | Serpentine 1 | Sep, 2015 |
|  | LMACI.0118/221/681 | LMAAI.0213 | Serpentine 2 | Oct, 2015 |
|  | LMACI.0543 | LMAAI.0524 | Serpentine 3 | Oct, 2015 |
|  | LMACI.0195 | LMAAI.0252 | Sumas 2 | Feb, 2016 |
|  | LMACI.0155 | LMAAI.0165 | Sumas 3 | Feb, 2016 |
|  |  |  |  | Jul, 2016 |
|  | LMACI.0122 (02-2448) | LMAAI.0003 | Serpentine 2 | Feb, 2016 |
|  | LMACI.0738 | LMAAI.1076 | Serpentine 2 | Mar, 2016 |
|  | LMACI.0044 (PNC_08-5757)/LMACI.0616 (ON_10PF0153) | LMAAI.0193/0818 | Serpentine 2 | Jul, 2016 |
|  | New | New | Serpentine 2 | Feb, 2016 |
|  | New | New | Serpentine 2 | Feb, 2016 |
| 1/2b | New | New | Sumas 1 | Nov, 2015 |
|  | New | LMAAI.0548 | Serpentine 2 | Dec, 2015 |
| 4b | LMACI.0003 (PNC_08-2076) | LMAAI.0019 | Serpentine 2 | Mar, 2015 |
|  |  |  | Serpentine 1 | Jan, 2016 |
|  | LMACI.0051 | LMAAI.0048 | Serpentine 2 | Apr, 2015 |
|  |  |  |  | Sep, 2015 |
|  |  |  |  | Feb, 2016^a^ |
|  |  |  |  | Feb, 2016^a^ |
|  |  |  | Serpentine 1 | Jan 2016 |
|  | LMACI.0071 | LMAAI.0022 | Serpentine 3 | Nov, 2015 |
|  | LMACI.0822 (PNC_15-0050) | LMAAI.1234 | Serpentine 2 | Dec, 2015 |
|  | LMACI.0009 (03-7263) | LMAAI.0112 | Serpentine 3 | Apr, 2016 |
| 4c | LMACI.0051 | LMAAI.0048 | Serpentine 1 | Sep, 2015 |

^a^These represent two different sampling dates within the same month.
